# Choroidal vascularity index of patients with coronary artery disease

**DOI:** 10.1038/s41598-022-07120-8

**Published:** 2022-02-22

**Authors:** Won-Woo Seo, Hyo Soon Yoo, Yong Dae Kim, Sung Pyo Park, Yong-Kyu Kim

**Affiliations:** 1 Division of Cardiology, Department of Internal Medicine, Hallym University College of Medicine, Kangdong Sacred Heart Hospital, #150 Seongan-ro, Gangdong-gu, Seoul, 05355 South Korea; 2 Department of Ophthalmology, Hallym University College of Medicine, Kangdong Sacred Heart Hospital, #150 Seongan-ro, Gangdong-gu, Seoul, 05355 South Korea; 3 Department of Otorhinolaryngology, Seoul National University Bundang Hospital, Seongnam, South Korea

**Keywords:** Cardiovascular diseases, Eye diseases, Diagnostic markers

## Abstract

We investigated the changes in subfoveal choroidal thickness and choroidal vascularity index (CVI) and their relationship with the severity of coronary artery stenosis in patients with cardiovascular risk factors and symptoms suggestive of coronary artery disease (CAD). Ninety patients who underwent coronary angiography (CAG) for evaluation of their coronary artery status and cardiac symptoms were included. Forty-two patients showed no evidence of CAD; 31 patients had one to two vessel disease; and 17 had a triple vessel disease. There were no significant differences in the subfoveal choroidal thickness among the three groups; however, the CVI in the triple vessel disease group was lower than those in the other groups. The CVI values were good predictors of the presence of triple-vessel disease (*p* = 0.020). Multivariate logistic regression analysis results revealed that male sex (odds ratio 5.4, *p* = 0.049), hypertension (odds ratio 4.9, *p* = 0.017), and CVI (%, odds ratio 0.8, *p* = 0.016) were significant factors associated with the presence of triple vessel disease. Although CVI may not be a sensitive marker for detecting early changes in the coronary artery, it may be helpful in indicating severe CAD.

## Introduction

Coronary artery disease (CAD) is one of the leading causes of death in the United States^1^. Catheter-based invasive coronary angiography (CAG) is the gold standard test for assessing the severity of CAD, however, the procedure is invasive with possible complications^2^. Therefore, various non-invasive modalities have been investigated, which indicate the severity of CAD^3^. In addition, several attempts have been made to determine the risk for cardiovascular diseases by assessing the changes in retinal blood vessels, which are easily accessible through non-invasive imaging tests. Several studies have shown that changes in retinal vessel diameters were associated with an increased risk of cardiovascular disease^4–6^. Furthermore, recent advances in imaging technology such as optical coherence tomography (OCT) have enabled examination of the choroid, a thin layer of a dense vascular network between the neural retina and the sclera. Most of the studies on choroid in patients with cardiovascular diseases assessed its thickness, and CAD patients were found to have thinner choroids than controls^7,8^.

Recently, a new quantitative parameter called the choroidal vascularity index (CVI) was introduced. CVI is a measure of the proportion of the luminal area, which corresponds to the vascular component, to the total choroidal area including both vascular and stromal components^9,10^. CVI is less variable and is influenced by fewer physiologic factors compared with choroidal thickness. Thus, CVI is a relatively stable index for studying the choroidal changes in various conditions^10^. In particular, choroidal analysis using CVI which represents both the luminal and stromal status of the choroidal vessels, rather than a simple measurement of the caliber of the retinal vessels, may be useful in confirming the hypothesis that the changes in blood vessels of the eye may reflect the degree of atherosclerosis in cardiovascular diseases. In this study, we aimed to investigate the changes in subfoveal choroidal thickness and CVI and their relationship with the severity of coronary artery stenosis in patients with cardiovascular risk factors and symptoms suggestive of CAD and who underwent CAG to evaluate their coronary artery status.

## Results

During the study period, 90 patients who underwent CAG for the coronary artery evaluation were enrolled. Ten patients were acute myocardial infarction, and 13 patients were suspected of unstable angina. Sixty-seven patients underwent CAG due to suspected stable angina, of which 38 patients were positive at treadmill tests. Forty-two patients showed no evidence of CAD including 3 patients with vasospastic angina, while 48 showed coronary artery narrowing or obstruction, suggesting CAD on CAG. The demographic and clinical characteristics and final clinical diagnosis after CAG of the patients are summarized in Table 1.

**Table 1:** Demographics and baseline clinical characteristics of patients with and without coronary artery disease.

	Group A No CAD (N = 42)	Group B 1–2 vessel disease (N = 31)	Group C Triple vessel disease (N = 17)	*P* value^a^	Post hoc anaylsis^b^
Age, yrs	61.6 ± 7.9	64.3 ± 9.6	66.5 ± 10.3	0.152	
Male, n(%)	24 (57)	22 (71)	15 (88)	0.062 / 0.019^c^	
Underlying diseases, n(%)					
Diabetes	8 (19)	4 (13)	8 (47)	0.020 / 0.066^c^	
Hypertension	14 (33)	17 (55)	13 (77)	0.008 / 0.002^c^	
Dyslipidemia or statin use	16 (38)	24 (77)	14 (82)	< 0.001 / < 0.001^c^	
Previous CAD	0	14 (45.2)	10 (58.8)	< 0.001 / < 0.001^c^	
BMI, kg/m^2^	25.4 ± 3.4	25.0 ± 3.7	25.8 ± 3.0	0.286	
Current smoker, n(%)	5 (12)	4 (13)	4 (24)	0.493 / 0.306^c^	
Medications, n(%)					
Calcium channel blocker	5 (12)	5 (16)	3 (18)	0.806 / 0.528^c^	
ARB/ACEi	8 (19)	12 (39)	4 (24)	0.163 / 0.406^c^	
Beta blocker	11 (26)	13 (42)	8 (47)	0.208 / 0.090^c^	
Nitrate	2 (5)	2 (7)	4 (24)	0.061 / 0.041^c^	
Final clinical diagnosis, n(%)				< 0.001 / < 0.001^c^	
Atypical chest pain	39 (93)	0	0		
Variant angina	3 (7)	1 (3)	0		
Stable angina	0	16 (52)	11 (65)		
Unstable angina	0	7 (23)	3 (18)		
Acute myocardial infarction	0	7 (23)	3 (18)		
Systolic BP, mmHg	115.6 ± 15.3	123.2 ± 21.4	122.9 ± 18.3	0.869	
Diastolic BP, mmHg	70.5 ± 11.9	75.9 ± 13.3	72.4 ± 12.0	0.491	
HbA1c, %	5.9 ± 0.8	6.2 ± 1.1	6.3 ± 1.2	0.565	
Total cholesterol, mg/dL	167.3 ± 41.9	146.3 ± 32.5	146.7 ± 39.3	0.046	A > B
HDL, mg/dL	50.8 ± 11.9	45.4 ± 10.2	42.6 ± 8.1	0.021	A > B, C
LDL, mg/dL	96.7 ± 27.3	83.0 ± 21.5	90.9 ± 29.3	0.089	
Triglycerides, mg/dL	138.2 ± 124.9	157.6 ± 103.1	133.1 ± 63.2	0.518	
Creatinine, mg/dL	0.84 ± 0.82	1.06 ± 1.25	0.84 ± 0.26	0.730	
eGFR, mL/min/1.73m^2^	95.0 ± 19.2	86.2 ± 25.3	88.8 ± 19.2	0.923	
Total leukocyte count, /mm^3^	6329 ± 2130	6998 ± 2508	7362 ± 2633	0.121	
Neutrophil count, /mm^3^	3485 ± 1214	4003 ± 1689	4236 ± 1740	0.271	
Lymphocyte count, /mm^3^	1946 ± 651	2136 ± 1421	1837 ± 730	0.791	
Monocyte count, /mm^3^	439 ± 172	487 ± 166	511 ± 169	0.231	
Neutrophil-to-lymphocyte ratio	1.94 ± 0.98	2.31 ± 1.47	2.61 ± 1.41	0.220	
Monocyte-to-lymphocyte ratio	0.24 ± 0.11	0.28 ± 0.15	0.30 ± 0.13	0.030	A < C
Ophthalmologic examination (Average of both eyes)					
BCVA, logMAR	0.15 ± 0.19	0.15 ± 0.13	0.16 ± 0.15	0.754	
IOP, mmHg	11.7 ± 3.1	11.3 ± 2.6	13.1 ± 3.4	0.069	
SE, diopters	0.3 ± 2.1	0.0 ± 1.8	0.6 ± 1.4	0.058	
Subfoveal choroidal thickness, μm	267.5 ± 93.1	233.7 ± 81.5	258.1 ± 103.9	0.264	
CVI without despeckling					
CVI_1500μm range	0.624 ± 0.030	0.626 ± 0.036	0.606 ± 0.037	0.127	
CVI_3000μm range	0.625 ± 0.027	0.627 ± 0.033	0.604 ± 0.037	0.040	A, B > C
CVI_5000μm range	0.625 ± 0.026	0.623 ± 0.033	0.606 ± 0.037	0.086	
CVI with despeckling					
CVI_1500μm range	0.652 ± 0.035	0.648 ± 0.041	0.629 ± 0.045	0.108	
CVI_3000μm range	0.652 ± 0.028	0.648 ± 0.035	0.626 ± 0.043	0.106	A > C
CVI_5000μm range	0.650 ± 0.027	0.643 ± 0.035	0.627 ± 0.041	0.057	A > C

^a^Kruskal-Wallis test and chi-square or Fisher’s exact tests were used to examine continuous and categorical variables, respectively.

^b^Post hoc analysis was performed using the Mann–Whitney U test.

^c^*P* value for trend using linear by linear association.

*ACEi* angiotensin-converting enzyme inhibitor, *ARB* angiotensin receptor blocker, *BCVA* best-corrected visual acuity, *BMI* body mass index, *BP* blood pressure, *CAD* coronary artery disease, *CVI* choroidal vascularity index, *eGFR* estimated glomerular filtration rate, *HDL* high-density lipoprotein, *IOP* intraocular pressure, *LDL* low-density lipoprotein, *logMAR* logarithm of the minimum angle of resolution, *SE* spherical equivalent.

There was no significant difference in age among the three groups; however, the greater the number of affected coronary vessels, the older the patients. Furthermore, a higher prevalence of CAD was associated with male sex, a history of hypertension, dyslipidemia or statin use, and nitrate use in patients with a higher number of affected coronary vessels. The no CAD group showed a higher level of total cholesterol compared to that of the 1–2 vessel disease group (*p* = 0.019, post hoc analysis using Mann–Whitney U test) and a higher level of high-density lipoprotein (HDL) compared to the other severe groups (No CAD vs. 1–2 vessel disease, *p* = 0.035; No CAD vs. Triple vessel disease, *p* = 0.015, post hoc analysis using Mann–Whitney U test). The triple vessel group showed a greater monocyte-to-lymphocyte ratio compared to the no CAD group (*p* = 0.007, post hoc analysis using Mann–Whitney U test, Table 1).

**Figure 1:**
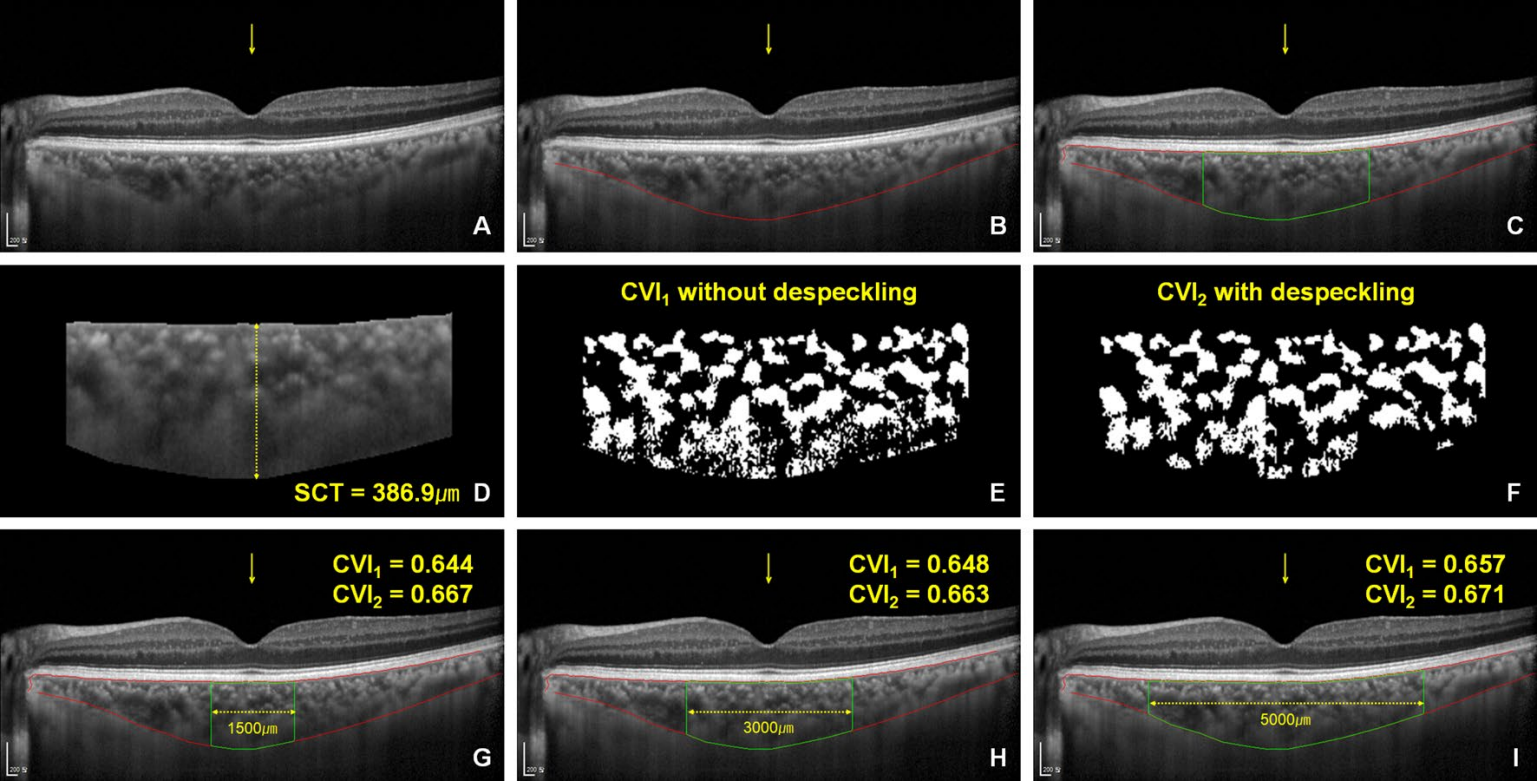
Choroidal vascularity index measurements. (**A**) The center of the fovea is marked with a built-in arrow tool (yellow arrow). (**B**) The outer boundary of the choroid is manually drawn (red line). (**C**) The lower border of the hyperreflective band of RPE/Bruch's membrane complex (upper red line) and the choroidal area of a predetermined range centered on the fovea are automatically extracted (green border area). (**D**) The subfoveal choroidal thickness (SCT) is automatically calculated. (**E**) Binarized images are obtained using the Niblack thresholding. (**F**) Despeckled image with the removal of particles with less than 20-pixels in size. (**G**–**I**) The choroidal vascularity index (CVI) is calculated as the ratio of the luminal area (dark area) to the total choroidal area in various choroidal ranges (1500 μm, 3000 μm, and 5000 μm) using both the original (CVI_1_) and despeckled (CVI_2_) binarized images.

Ophthalmic examinations revealed no significant differences in terms of visual acuity, intraocular pressure, and refractive errors among the three groups. CVI was measured in two different ways (with and without despeckling the binarized image) in three different scanning ranges (1500; 3000; and 5000 μm ranges, Fig. 1). There were no significant differences in the subfoveal choroidal thickness among the three groups; however, CVI was lower in the triple vessel disease group compared with those in the no CAD and 1–2 vessel disease groups in the 3000 μm and 5000 μm ranges (3000 μm range without despeckling: No CAD, 0.625 ± 0.027 vs. triple vessel disease, 0.604 ± 0.037, *p* = 0.046; 3000 μm range without despeckling: 1–2 vessel disease, 0.627 ± 0.033 vs. triple vessel disease, 0.604 ± 0.037, *p* = 0.020; 3,000 μm range with despeckling: no CAD, 0.652 ± 0.028 vs. triple vessel disease, 0.626 ± 0.043, *p* = 0.035; 5000 μm range with despeckling: no CAD, 0.650 ± 0.027 vs. triple vessel disease, 0.627 ± 0.041, *p* = 0.041; post hoc analysis using Mann–Whitney U test; Table 1).

**Figure 2:**
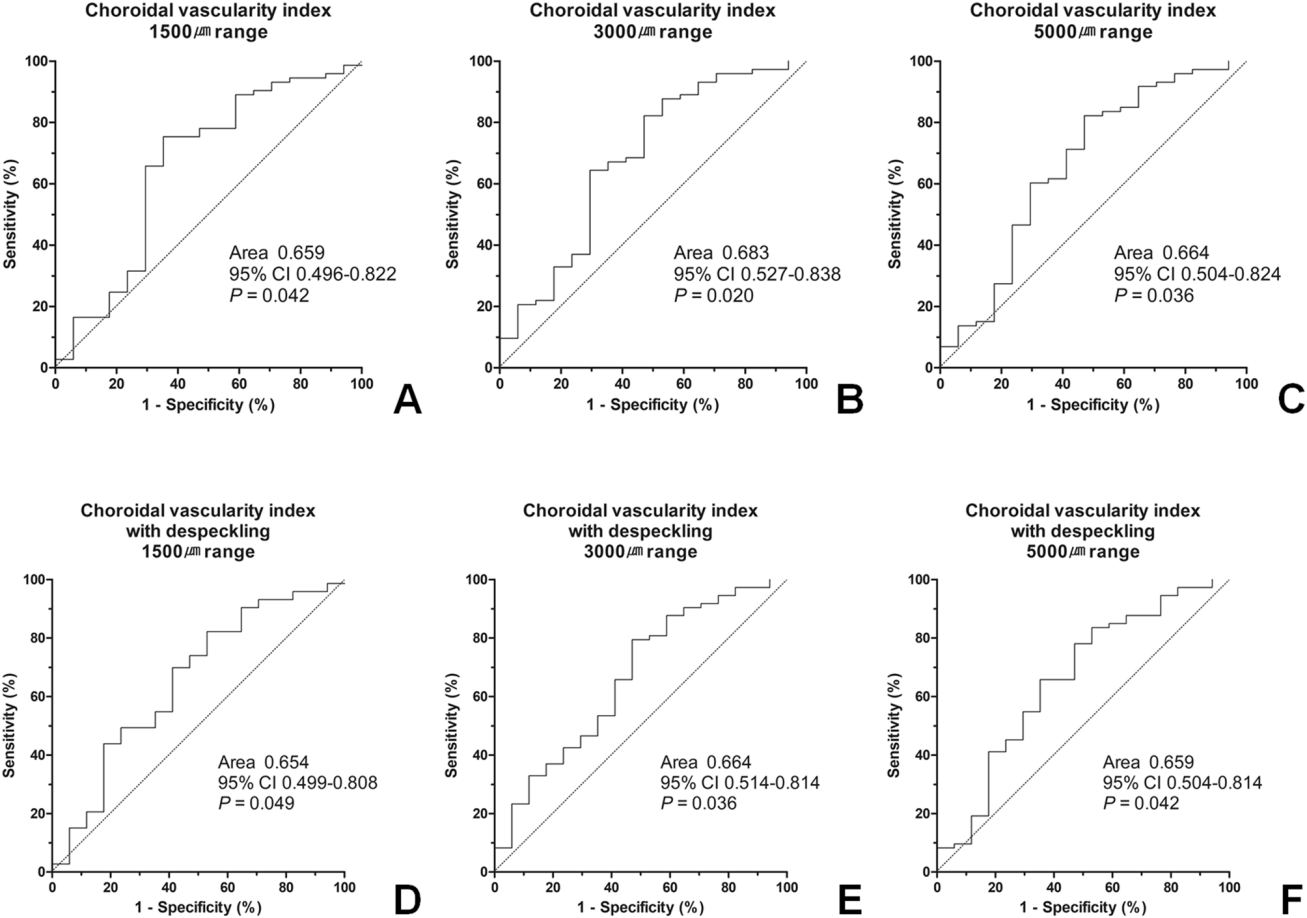
Receiver operating characteristic curve for predicting triple vessel disease using the choroidal vascularity index. The maximum sum of sensitivity and specificity was observed with CVI value cut-off points of 0.604, 0.601, and 0.600 for CVI without despeckling in 1500 μm, 3000 μm, and 5000 μm range analyses, respectively, and CVI value cut-off points of 0.624, 0.630, and 0.628 for CVI with despeckling in 1500 μm, 3000 μm, and 5000 μm range analyses, respectively. CI = confidence interval.

We evaluated whether CVI could predict the presence of CAD by analyzing the area under the receiver operating characteristic curve (AUC). The CVI value did not predict the presence of CAD (1500 μm range without despeckling: AUC 0.521, *p* = 0.728; 1500 μm range with despeckling: AUC 0.573, *p* = 0.235; 3000 μm range without despeckling: AUC 0.515, *p* = 0.802; 3000 μm range with despeckling: AUC 0.574, *p* = 0.228; 5000 μm range without despeckling: AUC 0.543, *p* = 0.482; 5000 μm range with despeckling: AUC 0.579, *p* = 0.199). However, the CVI values were good predictors of the presence of triple-vessel disease (1500 μm range without despeckling: AUC 0.659, *p* = 0.042; 1500 μm range with despeckling: AUC 0.654, *p* = 0.049; 3000 μm range without despeckling: AUC 0.683, *p* = 0.020; 3000 μm range with despeckling: AUC 0.664, *p* = 0.036; 5000 μm range without despeckling: AUC 0.664, *p* = 0.036; 5000 μm range with despeckling: AUC 0.659, *p* = 0.042, Fig. 2). The maximum sum of sensitivity and specificity was observed with CVI value cut-off points of 0.604, 0.601, and 0.600 for CVI without despeckling in 1500 μm, 3000 μm, and 5000 μm range analyses, respectively, and CVI value cut-off points of 0.624, 0.630, and 0.628 for CVI with despeckling in 1500 μm, 3000 μm, and 5000 μm range analyses, respectively.

On multivariate logistic regression analysis, male sex (odds ratio 3.4, *p* = 0.022) and dyslipidemia or statin use (odds ratio 7.3, *p* < 0.001) were significantly associated with the presence of CAD (Table 2). On the other hand, male sex (odds ratio 5.4, *p* = 0.049), history of hypertension (odds ratio 4.9, *p* = 0.017), and CVI (% in 3000 μm range without despeckling, odds ratio 0.8, *p* = 0.016) were significant factors associated with the presence of triple vessel disease (Table 3).

**Table 2:** Univariate and multivariate logistic regression analyses for factors associated with coronary artery disease.

Variables	Univariate analysis			Multivariate analysis		
	Odds ratio	95% CI	*P* value	Odds ratio	95% CI	*P* value
Age ≥ 65 years	2.4	1.0–5.6	0.049			
Male	2.5	1.02–6.3	0.046	3.4	1.2–9.4	0.022
Diabetes	1.4	0.5–3.9	0.499			
Hypertension	3.3	1.4–7.9	0.007			
Dyslipidemia or statin	6.2	2.4–15.7	< 0.001	7.3	2.7–19.7	< 0.001
CKD (eGFR < 60 mL/min/1. 73m^2^)	5.9	0.7–50.8	0.109			
Current smoker	1.5	0.4–4.9	0.523			
Obesity (BMI ≥ 25 kg/m^2^)	0.7	0.3–1.6	0.429			
NLR	1.4	1.0–2.1	0.082			
MLR	32.8	0.7–1657.3	0.081			
CVI_3000 μm (%)	0.9	0.8–1.1	0.375			

*BMI* body mass index, *CKD* chronic kidney disease, *CVI* choroidal vascularity index, *eGFR* estimated glomerular filtration rate, *MLR* monocyte-to-lymphocyte ratio, *NLR* neutrophil-to-lymphocyte ratio.

**Table 3:** Univariate and multivariate logistic regression analyses for factors associated with triple vessel disease.

Variables	Univariate analysis			Multivariate analysis		
	Odds ratio	95% CI	*P* value	Odds ratio	95% CI	*P* value
Age ≥ 65 years	2.8	0.9–8.4	0.068			
Male	4.4	0.9–20.7	0.061	5.4	1.01–29.2	0.049
Diabetes	4.5	1.5–14.1	0.009			
Hypertension	4.4	1.3–14.8	0.017	4.9	1.3–18.0	0.017
Dyslipidemia or statin	3.9	1.02–14.5	0.047			
CKD (eGFR < 60 mL/min/1.73m^2^)	1.8	0.3–10.3	0.501			
Current smoker	2.2	0.6–8.2	0.245			
Obesity (BMI ≥ 25 kg/m^2^)	1.4	0.5–4.2	0.498			
NLR	1.3	0.9–1.9	0.143			
MLR	13.3	0.4–492.6	0.161			
CVI_3000 μm (%)	0.8	0.7–0.97	0.018	0.8	0.7–0.96	0.016

*BMI* body mass index, *CKD* chronic kidney disease, *CVI* choroidal vascularity index, *eGFR* estimated glomerular filtration rate, *MLR* monocyte-to-lymphocyte ratio, *NLR* neutrophil-to-lymphocyte ratio.

On multiple linear regression analysis, the estimated glomerular filtration rate (eGFR; standardized β, 0.259; p = 0.011) and the presence of the triple vessel disease (standardized β, -0.254, *p* = 0.013) were significantly associated with CVI without despeckling measured in the 3000 μm range. In addition, eGFR (standardized β 0.242, *p* = 0.022), subfoveal choroidal thickness (standardized β 0.233, *p* = 0.027), and the presence of the triple vessel disease (standardized β -0.264, *p* = 0.007) were significantly associated with CVI with despeckling measured in the 3000 μm range (Table 4).

**Table 4:** Multiple linear regression analysis for factors associated with choroidal vascularity index measured on the 3000 μm range.

	Standardized β	Standard error	*p* value
**CVI without despeckling**			
eGFR, mL/min/1.73m^2^	0.259	0.015	0.011
Triple vessel disease	− 0.254	0.804	0.013
**CVI with despeckling**			
eGFR, mL/min/1.73m^2^	0.242	0.017	0.022
Subfoveal choroidal thickness, μm	0.233	0.004	0.027
Triple vessel disease	− 0.264	0.840	0.007

*CVI* choroidal vascularity index, *eGFR* estimated glomerular filtration rate.

## Discussion

In this study, we explored whether subfoveal choroidal thickness and CVI could reflect coronary artery status in patients with suspected CAD. There were no significant differences in subfoveal choroidal thickness and CVI between patients with and without CAD. However, CVI was lower in the triple vessel disease group than in the no CAD and 1–2 vessel disease groups.

Unlike the findings from previous studies^7,8^, there was no significant difference in subfoveal choroidal thickness in patients with different stages of CAD. This discrepancy may be explained by the heterogeneity in the populations examined. The patients in the no CAD group had no significant abnormalities in the coronary artery. However, they had risk factors and symptoms suggestive of CAD. These might have reduced the differences in choroidal thickness between patients with and without CAD. Intake of antihypertensive medications might also have contributed to the conflicting results. Although there were no significant differences in the incidence of CAD among groups who used various antihypertensive drugs, the incidence of CAD was highest among those who take nitrate.

Interestingly, CVI was decreased in the triple vessel disease group compared to the other stages of disease; however, no significant difference in CVI was observed between the no CAD group and the 1–2 vessel disease group. It might be difficult to sensitively detect the early progression of CAD through CVI. However, among the various traditional risk factors, only male sex, hypertension, and high CVI showed a significant association with triple-vessel disease. Dyslipidemia, which was significantly associated with the presence of CAD, did not show a significant association with the presence of triple-vessel disease. The patients included in this study are those with cardiac symptoms and needed CAG for accurate coronary artery evaluation. Our results suggest that CVI could be useful in differentiating severe CAD from less severe CAD.

After CVI was introduced by Agrawal et al.^10^, many researchers similarly measured CVI by binarizing the image of the subfoveal choroid of a particular area using various thresholding methods^11^. In particular, CVI values measured in different scan areas in the posterior pole were highly correlated^12^. On the other hand, CVI had a wide topographic variation when evaluated using wide-field optical coherence tomography (OCT)^13^. However, there is still a lack of consensus on optimal image binarization methods and scanning areas for CVI measurement. In this study, we measured the CVI using two different image binarization methods in three different areas. CVI, measured using each method, showed an overall similar tendency depending on the coronary artery status of the patient. However, the measurement in the 3000 μm range showed the largest AUC in the prediction of the presence of triple vessel disease. The 1500 μm range is a relatively narrow area, which may not be representative of the entire choroid. On the other hand, analysis that involves a wider area is likely to be affected by the shadowing of the retinal blood vessels^14^. Further research is needed on appropriate scanning areas that can reduce the variability of measurements and reflect the vascular status of the choroid.

We also used two different image binarization methods. First, the image was binarized using the Niblack auto-local thresholding method. The image was then despeckled to remove the presumed noise signal, especially those observed in the luminal area of the large vessels in the Haller’s layer. Both methods led to similar findings in predicting coronary artery status. However, in multiple linear regression analysis, CVI measured with the despeckled image showed a significant association with subfoveal choroidal thickness, while CVI measured using the binarized image without despeckling did not. Subfoveal choroidal thickness is associated with CVI, and the thicker the choroid, the greater the CVI^10^. However, in an eye with a thick choroid, the signal noise may increase since the light source has to penetrate the thick choroidal tissue, thereby decreasing the CVI. Therefore, despeckling the noise signal may be a useful way to adjust the bias. Nevertheless, further studies are needed to validate this finding.

The CVI also showed a significant association with the eGFR, which was consistent with previous findings. The choroidal thickness was associated with renal hemodynamics, which was assessed via Doppler sonography^15^. The subfoveal choroidal thickness was significantly correlated with renal function, which was measured using the eGFR^16–18^. In a study on choroidal thickness and CVI changes following hemodialysis in patients with end-stage renal disease, the choroidal thickness was significantly decreased after hemodialysis, while the CVI value did not change much. These findings suggest that CVI may be a more stable index for vascular status compared with choroidal thickness^19^. In our study, CVI was associated with the microvascular status of the two major organs, the heart and kidney.

Our study has several limitations. First, our sample size was small and not evenly distributed among three different CAD severity groups. Although we performed multivariate analysis to reduce the confounding effects of the variables, there were significant differences in terms of underlying diseases such as diabetes and hypertension, among different groups which are known to be associated with a choroidal thickness or CVI values^20–22^. Second, although those in the no CAD group did not have significant coronary stenosis, there was no healthy control group without cardiovascular risk factors or symptoms. Third, in this study, we could only investigate the association between the severity of CAD evaluated by CAG and CVI, however, we were not able to examine the relationship between clinical cardiac symptoms or diagnosis and CVI in detail. Fourth, we need to optimize the method in measuring CVI consistently as a biomarker of systemic vascular status.

In conclusion, CVI was reduced in patients with triple-vessel disease compared with that in patients with low stages of CAD. The cut-off values of approximately 0.630 and 0.600 for CVI values measured using binarized images with and without noise despeckling, respectively, could be used to predict the risk of triple vessel disease. Although CVI may not be a sensitive indicator of early changes in the coronary artery, it can help suggest severe coronary artery obstruction.

## Methods

### Participants

Patients who underwent CAG for the evaluation of their cardiac symptoms were recruited between September 1, 2017, and February 28, 2019. The inclusion criteria were as follows: (1) age between 20 and 80 years and (2) those with cardiac symptoms suggestive of CAD who subsequently underwent CAG. The exclusion criteria were as follows: (1) those with chorioretinal disorders, including diabetic retinopathy, age-related macular degeneration, retinal vein occlusion, retinal artery occlusion, central serous chorioretinopathy, and uveitis; (2) those with severe cataract, corneal disease, or media opacity that obscured the clear OCT image acquisition; and (3) those who underwent intraocular surgery except for uncomplicated cataract surgery. The study was approved by the Institutional Review Board (IRB) of the Kangdong Sacred Heart Hospital (IRB No. 2017–07-004). All study conduct adhered to the tenets of the Declaration of Helsinki, and written informed consent was obtained from all study participants after an explanation of the nature and possible consequences of the study.

### Coronary angiography and ophthalmologic examinations

CAG was performed using the radial or femoral artery approach in all patients. Coronary lesion severity was independently evaluated by two experienced cardiologists. They used the visual estimation method on multiple orthogonal angiographic views. We defined significantly obstructed CAD as ≥ 50% of the luminal diameter narrowing in one or more major epicardial coronary arteries or the presence of coronary stents due to previous coronary intervention.

All patients underwent routine ophthalmologic examinations, including slit-lamp examination, intraocular pressure measurement, and fundoscopy examination. The best-corrected visual acuities were measured using the Snellen chart and converted to the logarithm of the minimal angle of resolution for analysis. Refractive errors were evaluated using an auto-kerato-refractometer (KR-8900, Topcon Corporation, Tokyo, Japan). The patients underwent indirect ophthalmoscopic examination, wide-field fundus photography (Optos 200TX, Optos PLC, Dunfermline, Scotland), and OCT (Spectralis OCT, Heidelberg Engineering, Heidelberg, Germany).

### Choroidal vascularity index analysis

The choroidal vascularity index was semi-automatically calculated. First, we marked the center of the fovea using a built-in arrow tool in a horizontal enhanced depth imaging (EDI) OCT image (Fig. 1A). The boundary between the choroid and sclera was manually drawn by an experienced examiner (Y-K.K.) using the segmented line tool in ImageJ (National Institutes of Health, Bethesda, MD, USA) (Fig. 1B). The lower border of the hyperreflective band of the retinal pigment epithelium (RPE)/Bruch’s membrane complex (Fig. 1C, upper red line) and choroidal area of a predetermined range centered on the fovea were automatically extracted (Fig. 1C, green border area). If the automatically extracted RPE border was inaccurate, we manually drew the RPE border using the segmented line tool in ImageJ. The subfoveal choroidal thickness was automatically calculated as the distance between the RPE and the choroidal borders at the center of the fovea (Fig. 1D). The whole OCT image was binarized using the Niblack method, and the binarized image of the predetermined choroidal range was evaluated. The pixel was thresholded by the surrounding 25 × 25 neighborhood pixel intensity information using the equation pixel intensity > mean intensity + 0.1 × standard deviation (Fig. 1E). The image was despeckled by removing particles that were less than 20-pixel in size (Fig. 1F). The CVI was calculated as the ratio of the luminal area (dark area) to the total choroidal area using the two types of images (original binarized images and despeckled images) in various choroidal ranges (1,500; 3,000; and 5,000 μm range; Fig. 1G–I) . The average value of both eyes was used for evaluation. All analyses were performed using the MATLAB version R2020b. All MATLAB scripts used for the analysis are provided at GitHub (https://github.com/ykkim7/Choroidal-vascularity-index).

### Statistical analyses

We classified patients into three groups according to the number of obstructed coronary arteries confirmed by CAG. The three groups were as follows: no CAD, 1–2 vessel disease, and triple vessel disease. We compared the clinical characteristics, such as underlying diseases, body mass index (BMI), smoking, hypertensive medications, blood pressure, glycated hemoglobin (HbA1c), cholesterol, creatinine, eGFR, and leukocyte counts including neutrophils, lymphocytes, monocytes, and neutrophil-to-lymphocyte ratio (NLR) and monocyte-to-lymphocyte ratio (MLR), among the three groups. As the observed variables did not meet the normality distribution assumption assessed by the Kolmogorov–Smirnov test, the nonparametric Kruskal–Wallis test with post hoc analysis using Mann Whitney U test was used for the comparison of continuous variables. Chi-square or Fisher’s exact tests were used for the comparison of categorical variables. We performed univariate and multivariate logistic regression analyses to explore the factors associated with CAD or triple-vessel disease. We explored the possible risk factors using univariate analysis and performed multivariate analysis using the variables with p values less than 0.1. Significant factors were selected using the stepwise regression. We performed the receiver operating characteristic (ROC) curve analysis to predict CAD or triple-vessel disease using the CVI. Statistical analyses were performed using IBM SPSS version 23.0 (IBM Corp., Armonk, NY, USA). Statistical significance was defined as a p-value < 0.05.

## Data Availability

The datasets generated during the current study are available from the corresponding author upon request.
